# *Lactobacillus gasseri* CRISPR-Cas9 characterization *In Vitro* reveals a flexible mode of protospacer-adjacent motif recognition

**DOI:** 10.1371/journal.pone.0192181

**Published:** 2018-02-02

**Authors:** Emily M. Anderson, Shawn McClelland, Elena Maksimova, Žaklina Strezoska, Megan Basila, Alexandra E. Briner, Rodolphe Barrangou, Anja van Brabant Smith

**Affiliations:** 1 Dharmacon, a Horizon Discovery Group Company, Lafayette, CO, United States of America; 2 Department of Food, Bioprocessing and Nutrition Sciences, North Carolina State University, Raleigh, NC, United States of America; New England Biolabs Inc, UNITED STATES

## Abstract

While the CRISPR-Cas9 system from *S*. *pyogenes* is a powerful genome engineering tool, additional programmed nucleases would enable added flexibility in targeting space and multiplexing. Here, we characterized a CRISPR-Cas9 system from *L*. *gasseri* and found that it has modest activity in a cell-free lysate assay but no activity in mammalian cells even when altering promoter, position of tag sequences and NLS, and length of crRNA:tracrRNA. In the lysate assay we tested over 400 sequential crRNA target sequences and found that the Lga Cas9 PAM is NNGA/NDRA, different than NTAA predicted from the native bacterial host. In addition, we found multiple instances of consecutive crRNA target sites, indicating flexibility in either PAM sequence or distance from the crRNA target site. This work highlights the need for characterization of new CRISPR systems and highlights the non-triviality of porting them into eukaryotes as gene editing tools.

## Introduction

The CRISPR-Cas9 (Clustered Regularly Interspaced Palindromic Repeats-CRISPR-associated protein 9) system, derived from a bacterial immune system pathway, has emerged as a dominant platform for gene editing in a host of applications, especially in mammalian cells [1–3]. The Cas9 nuclease can be directed to targets by loading it with two small RNAs (transactivating CRISPR-associated RNA (tracrRNA) and the target-specific CRISPR RNA (crRNA)) that enable the formation of double-stranded breaks (DSBs) at almost any desired sequence without having to re-engineer the Cas9 protein, a requirement of other site-specific nucleases [4, 5]. Upon resolution of the DSB by endogenous cellular mechanisms, genes can be knocked out or their function modulated in precise way by introduction of mutations, tags, or other sequence edits [6, 7].

In addition to a 20-nucleotide (nt) region of complementarity between a targeting crRNA and desired target site, a short protospacer-adjacent motif (PAM) [8] is required downstream of the target DNA on the non-targeted strand. For the most commonly used *Streptococcus pyogenes* (Spy) CRISPR-Cas9, the PAM is NGG [9, 10]. This short, well-defined PAM has enabled Spy Cas9 to be broadly applied for gene editing applications requiring gene knockout, including large scale or genome scale screening applications [11–17]. On average the NGG PAM can be found every 8 nucleotides in the human genome [1].

In addition to widespread use of Spy Cas9, efforts have been made toward discovery of additional Cas9s/Type II effector nucleases such as Cas12a/Cpf1 and Cas12b/C2c1 [18–21]. Different RNA-programmed editing nucleases could provide benefits including increased nuclease activity or specificity, a smaller Cas9 for viral packaging and delivery, and additional PAM sequences to allow flexible and specific targeting capacity (for instance in AT-rich regions) as well as orthogonal use of multiple Cas9s.

Here, we have isolated and characterized a Type II-A CRISPR-Cas9 from a strain of *Lactobacillus gasseri* (Lga), a nonpathogenic and commensal bacterium originally isolated from human mucosal tissues with a history of safe human consumption as a probiotic. It has been previously reported that Lga contains a signature Cas9 gene, as well as a tracrRNA and CRISPR array [22]. Native CRISPR activity was demonstrated by a plasmid interference assay against targets possessing the bioinformatically predicted PAM of 5’-NTAA-3’, but not a mutated PAM of 5’-GCTC-3’. A short, well-defined PAM of completely orthogonal sequence to that of Spy (NTAA versus NGG, S1 Table) made this an interesting system to investigate.

We codon-optimized the Lga Cas9 gene for mammalian expression and cloned it into mammalian expression vectors. While it exhibited modest RNA-directed DNA cleavage activity when expressed and tested in an *in vitro* lysate assay, we could not obtain insertion nor deletion (indel) formation when transfected into mammalian cells with synthetic crRNA and tracrRNA. Configurations of the vector, including promoters and positions of sequence tags and nuclear localization sequences (NLS), did not improve enzymatic activity. Using the *in vitro* cleavage assay with PCR amplicon targets, we performed a high-throughput survey of over 400 crRNAs to characterize the required PAM sequence, which surprisingly was found to be represented by NNGA/NDRA (different than the predicted bacterial NTAA). In addition, there were multiple instances of Lga Cas9 targeting sequential sequences, indicating flexibility in the PAM sequence or position not observed in other published CRISPR-Cas9 systems.

## Results

**Fig 1:**
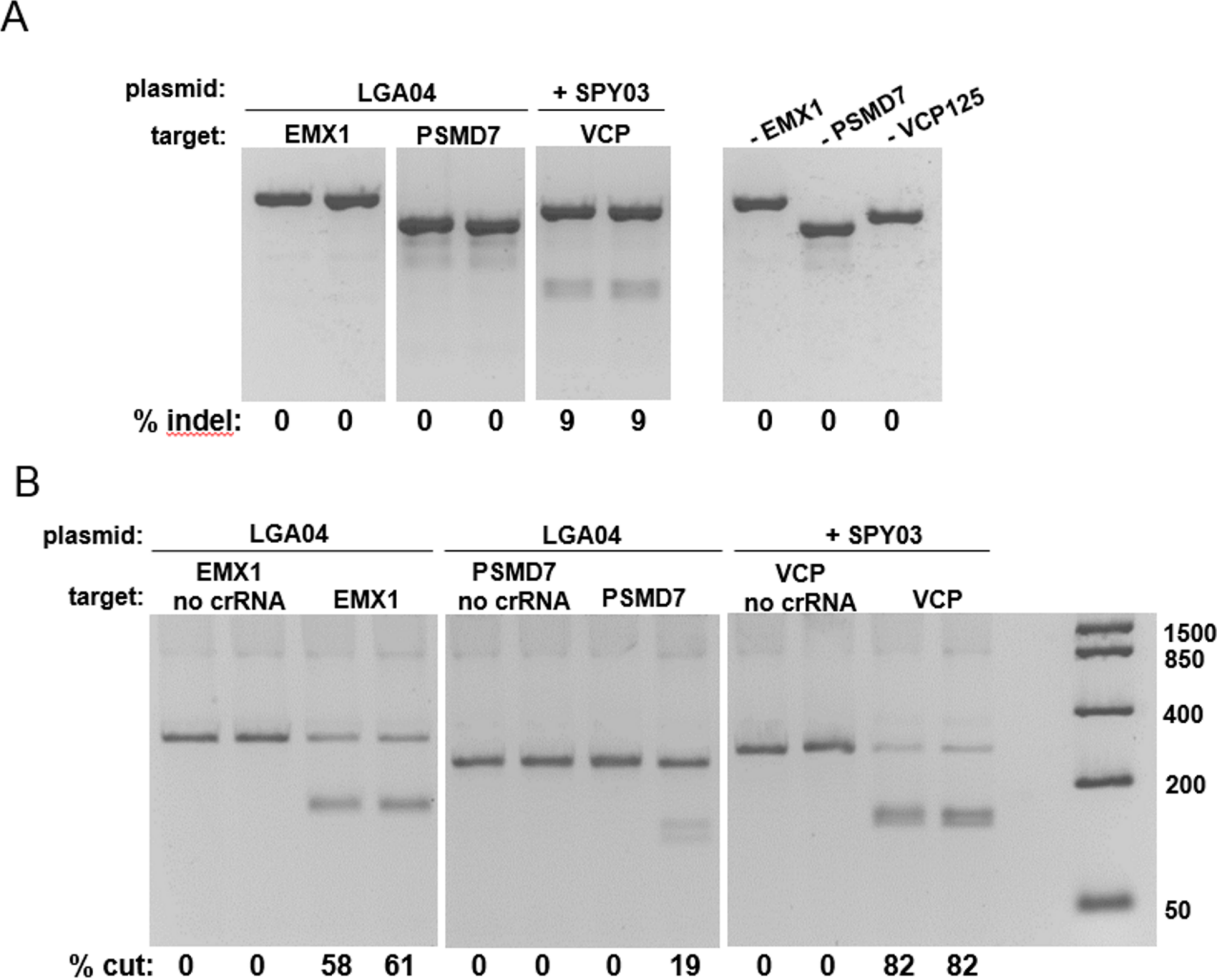
Examination of the in-cell editing and *in vitro* activity of Lga Cas9 nuclease. (A) The T7EI mismatch assay for detection of in-cell gene editing activity upon codelivery of Lga Cas9 plasmids and crRNA:tracrRNA against two target sites in the EMX1 and PSMD7 genes (D and W59 respectively). Spy Cas9 and crRNA:tracrRNA targeting VCP (#125) was used as a reference control. Amplicons from untransfected cells for the three target sites were used as negative controls in the T7EI assay. Percent indel formation is shown at the bottom of the gels. (B) DNA cleavage assay for detection of in *vitro* activity of Lga Cas9. Cell lysate, generated from cells transfected with either Lga Cas9 expression plasmid (LGA04) or Spy Cas9 expression plasmid (SPY03) were used to determine the ability to cut the DNA amplicons containing the same target sites in EMX1, PSMD7 or VCP1 as in 1A. Percent cutting is shown at the bottom of the gels.

To test for expression of Lga Cas9, plasmids containing FLAG-tagged Lga Cas9 were transfected into HEK293T cells, and relative protein expression was determined by Western Blot. The expression of Lga Cas9 was as high or higher than a control plasmid expressing Spy Cas9 for all three vectors with different configurations of the FLAG tag position and linker length (S1 Fig). To assess gene editing activity in cells, co-transfections were performed with a plasmid expressing untagged Lga Cas9and synthetic crRNA:tracrRNA targeting human genes (EMX1 and PSMD7) containing the predicted NTAA PAM, again compared to transfection of a control Spy Cas9plasmid and crRNA:tracrRNA targeting VCP (with NGG PAM). A purification tag was not included, as in previous work with the Spy Cas9, inclusion of a tag in some locations (N-terminal versus C-terminal, etc.) inhibited activity to some extent. A mismatch detection assay (T7EI) was used to determine gene editing activity in the cell population. In parallel, plasmids were transfected into cells without crRNA:tracrRNA to prepare lysate for an *in vitro* assay [19]. The T7EI assay (Fig 1A) indicated no gene editing on the Lga Cas9 target sites, while Spy Cas9 displayed modest levels of activity (9%). In Fig 1B, the *in vitro* assay measured strong activity of the Lga Cas9 toward these same target sites in DNA amplicons, however still lower than that obtained from the Spy lysate (~20 to 60% cut amplicon versus 82%). In subsequent *in vitro* experiments, activity of the Lga Cas9 toward a handful of additional target sites revealed that in general, target cleavage was in the range of 10–20%, with the EMX1 target site being an exceptionally active site (data not shown). The range of Lga *in vitro* activity while being unable to produce gene editing activity in cell culture, compared to Spy which had in-cell activity for all crRNAs tested, suggests that selection of a weak crRNA is not the primary reason for Lga Cas9 lack of activity *in vivo*.

**Fig 2:**
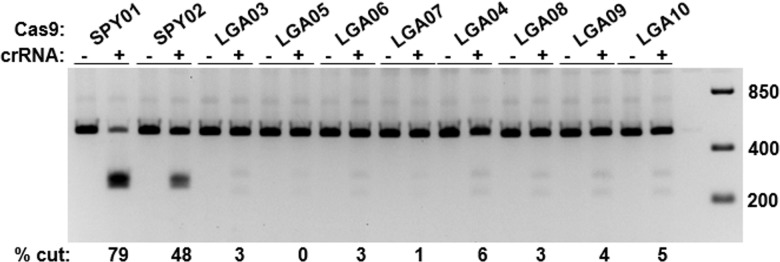
*In vitro* activity of Lga Cas9 protein expressed from various vectors. An *in vitro* DNA cleavage assay was performed against the PSMD7 target amplicon to assess the activity of lysates from cells transfected with the different Lga Cas9 vector constructs. Spy Cas9 lysate and the VCP amplicon was used as reference control. Percent cutting is indicated at the bottom of the lanes.

It was unclear why Lga Cas9 with crRNA:tracrRNA could not effect targeted gene editing in cells and exhibited lower cutting activity than Spy Cas9 *in vitro*. To test whether activity was negatively affected by vector design, various configurations were constructed (S2 Fig). Activity of Lga Cas9 protein expressed from the different vector constructs was examined in the *in vitro* assay and was again significantly lower than activity of Spy Cas9 lysate toward respective cognate target sites (both Lga and Spy sites residing in the same PSMD7 amplicon, Fig 2). Lga Cas9 activity was slightly higher when expressed from the CMV promoter, and when either the NLS or FLAG tag were inserted tagging the C-terminus. However, in vitro activity against this target site was generally low enough that these slight differences could be within error and detection limit of the assay.

**Fig 3:**
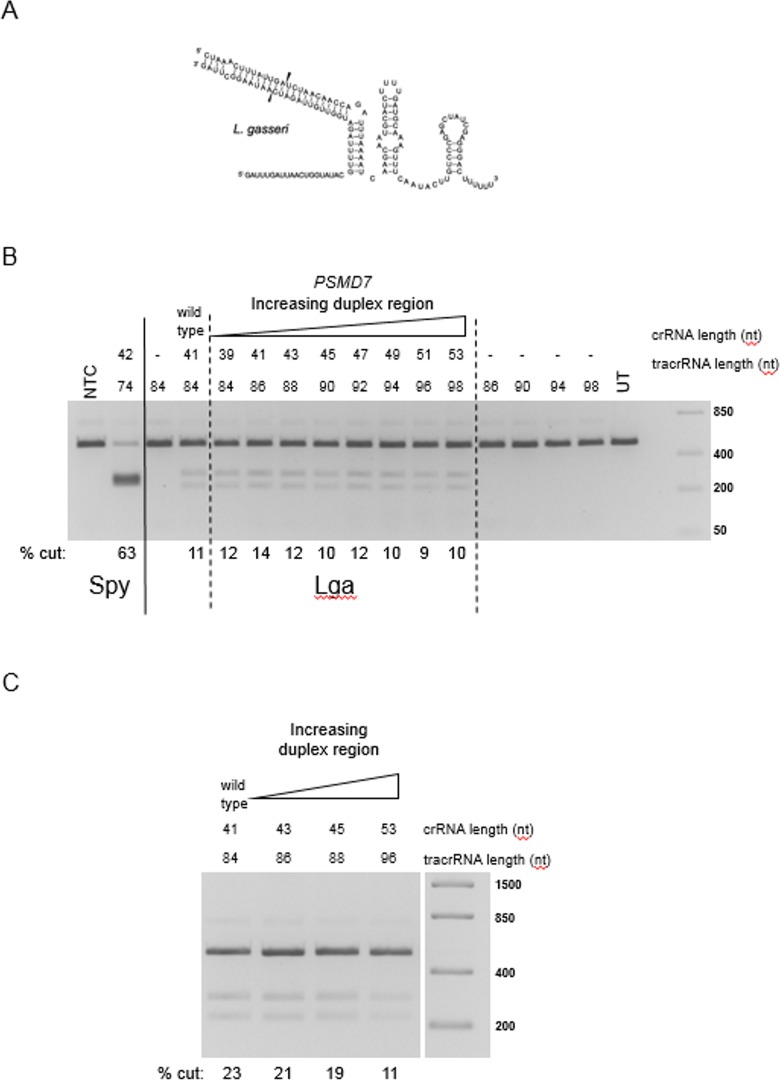
Effect of increasing the lengths of Lga crRNA:tracrRNA repeat-anti-repeat region on Lga cleavage activity. (A) Predicted RNA secondary structure of Lga crRNA complexed with tracrRNA. The predicted Lga guide RNA contains a 41 nt crRNA sequence and an 84 nt tracrRNA (wild type) with a 2 nucleotide 3’ overhang on the crRNA. The predicted RNAse III cut sites which generate the mature crRNA and tracrRNA are indicated by arrows. (B) The predicted Lga crRNA:tracrRNA sequence (wild type) targeting *PSMD7* was compared to longer crRNA:tracrRNA complexes in an *in vitro* cleavage assay where there is no 3’ overhang on the crRNA with respect to the tracrRNA. (C) The predicted Lga crRNA:tracrRNA sequence (wild type) targeting *PSMD7* was compared to the longer crRNA:tracrRNA complexes in an *in vitro* cleavage assay where there is a 3’ overhang on the crRNA with respect to the tracrRNA.

To test that the Lga crRNA:tracrRNA sequence used was not sufficiently long or of the correct secondary structure to load Cas9 and produce activity, experiments with various lengths of the small RNAs were conducted. In the native bacterium, it may be processed to a longer form than originally predicted by an RNase III cut site analogous to the Spy system. First, the 3ʹ end of the crRNA and the 5ʹ end of the tracrRNA were lengthened in a coordinate manner in a series of 2 nt increments (crRNA from 39 nt to 53 nt, Fig 3A and S2 Table). These crRNA:tracrRNAs were tested both as blunt-ended and containing a 2nt 3ʹ overhang on the crRNA. None of the lengthened crRNA:tracrRNA complexes significantly improved Cas9 activity *in vitro* (Fig 3B and 3C).

Next, we interrogated whether the predicted Lga PAM was adequate for target site selection in a gene editing context, versus its use in the native bacterial host for genome defense. It is possible that target sites initially chosen did not elicit a high degree of Cas9 activity due to sub-optimal sequence composition. It is also possible that the PAM consensus derived from characterization of the Lga Cas9 in the native bacterium does not describe ideal PAM recognition when the enzyme is expressed in a non-native context, such as in mammalian cells. To examine these possibilities, we used the *in vitro* assay in a high-throughput manner to expand testing of potential Lga Cas9 target sites.

A high-throughput PAM Assay was designed by “walking” crRNAs in one nt increments across one strand of a target amplicon (agnostic of the PAM sequence, not restricting the PAM to NGAA motifs) where the cut bands could be distinguished on an agarose gel. Lga Cas9 crRNAs were synthesized for amplicons derived from three genes (S3 Fig). A total of 446 crRNA sequences were tested, sampling all sixty-four possible three-base PAMs at least once, and many cases several 20mer target sites for a given PAM. If the potential PAM motif is extended to a length of four bases (256 sequences) or five bases (1024 sequences), coverage of 74% and 33% of all possible permutations of PAM motifs is achieved, respectively.

**Fig 4:**
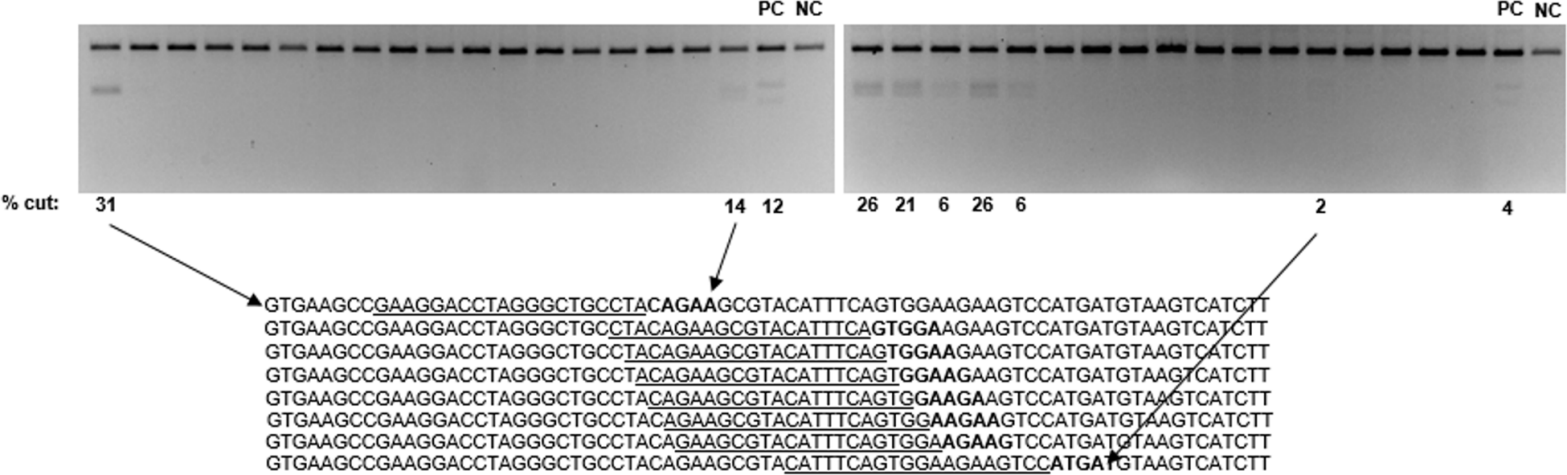
Representative agarose gel images from the *in vitro* PAM walk experiment conducted with the PSMD7 gene target. The percent cutting for active sequences is indicated below the gel. (PC) positive control. (NC) negative control. The target sequence and surrounding sequence is indicated for both isolated active sites as well as an example of consecutive active target sites. Target sites are underlined for all active sequences and for some, are paired with their lane on the gel for orientation. The percent cutting measurement can only be interpreted as a semi-quantitative estimate due to small differences in lysate batches in the *in vitro* assay measurement, as well as staining differences in gels. Comparisons of the efficacy of one target site/PAM versus another are not made except to conclude whether or not they have cutting activity.

As expected if a PAM were required for initial Cas9 binding and licensing of nuclease activity [9], only a fraction of sites (41/446 or 9%) displayed activity, as most sequences are not adjacent to a predicted PAM (Fig 4, S3 Table). Interestingly, many of the PAMs associated with active crRNAs did not match the NTAA sequence predicted for Lga Cas9. Surprisingly, there were five instances of consecutive crRNA cleavage “hits” (up to 6 in a row, Fig 4). In context of the low hit percentage overall, this is an extremely high rate of consecutive crRNA hits. Other Cas9s have a positionally defined PAM sequence immediately adjacent to the target sequence (such as the 3ʹ non-cognate strand NGG PAM for Spy Cas9). The PAM walk data for Lga suggest two conclusions: the Lga PAM sequence may be more flexible or degenerate than originally predicted, and since there are multiple cases of adjacent active crRNA target sites, there could be a flexible length between the PAM motif and the crRNA target in certain sequence contexts.

**Fig 5:**
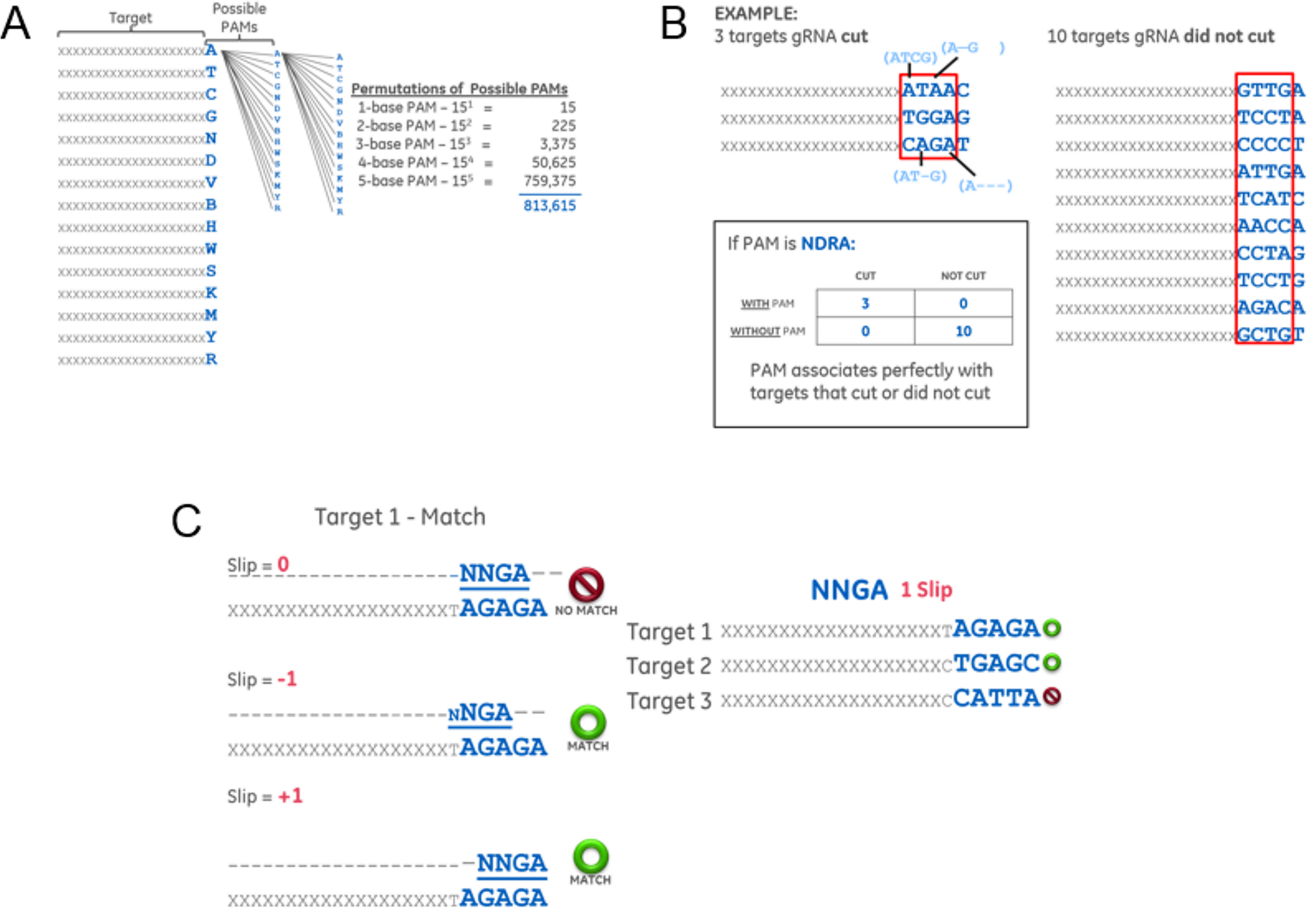
PAM analysis method. (A) A diagram illustrating the generation of all possible PAM sequence motifs for lengths of one to five bases, including degenerate base designations, to be compared against the PAM sequences identified in the PAM walk experiment. The one-letter codes for bases follow standard IUPAC nomenclature: A, T, C, G = standard DNA bases adenine, thymine, cytosine, and gunaine; N = any of the four standard DNA bases; D = A, G, T; V = A, C, G; B = C, G, T; H = A, C, T; W = A, T; S = G, C; K = G, T; M = A, C; Y = C, T; R = A, G. (B) Diagram illustrating the method of scoring the lists of PAM sequences of targets site that were cut or not cut by Lga Cas9 against each potential PAM motif. The example provided uses the four-base PAM NDRA. (C) Diagram illustrating how potential PAM sequence motifs were aligned to the PAM sequences tested in the PAM walk experiment to allow for the targeting region of the crRNA to slip either one base forward or backward before cutting at the DNA target location indicated by the 20mer guide sequence of the crRNA.

For each possible permutation of PAM motif up to a length of 5 nucleotides, analysis was performed to determine the number of tested crRNAs that fit one of four descriptions (Fig 5B). A Fisher’s exact test was used to determine the level to which a given PAM motif is discriminating crRNAs that cut versus those that do not cut. To analyze all possible PAM motif permutations, all degenerate base designations were included in the analysis. Over 800,000 PAM motifs of length one to five were ranked by Fisher’s exact test p-value to determine a predictive PAM (Fig 5A and 5B). If only the standard determinate bases A, G, C, and T are considered (and include N as a spacer), the strongest PAM is NNGA (Table 1). If all degenerate bases are included, the strongest PAMs to emerge are NDRA or NNRA (Table 2).

**Table 1:** Strongest PAMs considering only standard DNA base designations.

PAM Length	PAM	# Targets that DO Cut		# Targets that DO NOT Cut		Fisher’s Exact Test p-value
		WITH PAM	WITHOUT PAM	WITH PAM	WITHOUT PAM	
5	NNGAN	22	19	7	398	2.77E-20
4	NNGA	22	19	7	398	2.77E-20
4	NNNA	30	11	69	336	2.21E-13
5	NNNAN	30	11	69	336	2.21E-13
5	NNGNN	29	12	81	324	6.47E-11
4	NNGN	29	12	81	324	6.47E-11
3	NNG	29	12	81	324	6.47E-11
4	NTGA	10	31	2	403	8.35E-10
5	NTGAN	10	31	2	403	8.35E-10
5	ANGAN	9	32	1	404	1.84E-09

**Table 2:** Strongest PAMs including degenerate base designations.

PAM Length	PAM	# Targets that DO Cut		# Targets that DO NOT Cut		Fisher’s Exact Test p-value
		WITH PAM	WITHOUT PAM	WITH PAM	WITHOUT PAM	
4	NDRA	29	12	14	391	1.37E-25
5	NDRAN	29	12	14	391	1.37E-25
5	NNRAN	30	11	18	387	3.42E-25
4	NNRA	30	11	18	387	3.42E-25
5	DDRAN	27	14	10	395	6.30E-25
4	DDRA	27	14	10	395	6.30E-25
4	DNRA	27	14	12	393	6.61E-24
5	DNRAN	27	14	12	393	6.61E-24
4	DDRR	34	7	39	366	7.59E-24
5	DDRRN	34	7	39	366	7.59E-24

The hypothesis that the distance between the PAM motif and the 20 nt target might be flexible prompted us to analyze each motif again, but allowing for the motif to slip or shift forward, backward, or both. For example, for a crRNA target site 5ʹ of a PAM sequence of AGAGA we could allow a NNGA motif with a shift of +1 or -1. This analysis, whether we allowed for both a +1 and -1 shift or each shift individually, in each case established the NNGA motif as strongest (Fig 5C and Table 3).

**Table 3:** Strongest PAMs allowing for PAM positional shift.

PAM Length	PAM	# Targets that DO Cut		# Targets that DO NOT Cut		Fisher’s Exact Test p-value
		WITH PAM	WITHOUT PAM	WITH PAM	WITHOUT PAM	
**standard bases with shift +/- 1**						
4	NNGA	31	10	66	339	7.88E-15
5	NNGAN	31	10	66	339	7.88E-15
5	NNGAA	10	31	4	401	1.11E-08
4	NNNA	38	3	219	186	4.68E-07
5	NNNAN	38	3	219	186	4.68E-07
**standard bases with shift +1**						
4	NNGA	27	14	38	367	9.93E-16
5	NNGAN	27	14	38	367	9.93E-16
5	NNNAN	35	6	151	254	2.11E-09
4	NNNA	35	6	151	254	2.11E-09
5	NNGAA	9	32	2	403	9.48E-09
**standard bases with shift -1**						
4	NNGA	26	15	40	365	2.69E-14
5	NNGAN	26	15	40	365	2.69E-14
5	NNGAA	9	32	2	403	9.48E-09
4	NNNA	33	8	151	254	1.39E-07
5	NNNAN	33	8	151	254	1.39E-07

The degenerate motif NNRA could be explained equally well by a shift, or flexible distance, of an actual more proscribed non-degenerate base PAM. An NNGA motif supplemented by a -1 shift to a NGA motif would allow a purine R (A or G) at the 3^rd^ nucleotide. The very strong p-value obtained for the degenerate NNRA motif could, in fact, be describing the non-degenerate NNGA motif with some flexibility of the length between the target sequence and the PAM (Tables 2 and 3).

Whether these results are interpreted as a degenerate PAM of NNRA/NDRA or a flexible length/distance PAM of the more defined NNGA, this identified PAM is different from the PAM originally identified for Lga Cas9 in the native bacterium (NTAA). In the *in vitro* assay, only the minimal components for Cas9-directed double-strand break nuclease activity were present, namely the Cas9 nuclease, tracrRNA, and a targeting crRNA. It could be that within the intact CRISPR system in *L*. *gasseri*, other proteins involved in the adaptation and expression phases could influence which PAM sequence is optimal for a functional CRISPR immune response, which is reflected in the consensus PAM described from the comparison of the sequenced bacterial crRNA arrays and the sequenced viral invader genomes.

## Discussion

We have expressed the Lga Cas9 enzyme in mammalian cells to demonstrate that the protein in a cell-free extract can be combined with crRNA:tracrRNA to cleave DNA targets in an *in vitro* assay. Lga Cas9 did not exhibit as robust activity as Spy Cas9 when compared across multiple targets, and we were unable to elicit indel formation upon transfection of Lga Cas9 with crRNA:tracrRNA in cells. The expression context as well as the length and secondary structure of the crRNA:tracrRNA duplex region did not affect activity of the expressed enzyme. This is not unprecedented compared to recent reports characterizing other Class II CRISPR effector nucleases: Type IIA/C Cas9 orthologs [23], Cas12a/Cpf1 nucleases from various species [21], and the Cas12b/C2c1 nuclease [19]. While it is not specified in all of these examples why many nucleases exhibit robust activity *in vitro* but not in cells, some explanations could include: lack of a required protein or RNA cofactor from the native bacterium, inability of a given Cas9 enzyme to efficiently unwind target DNA in the mammalian cellular context of methylation/nucleosome packaging, sub-optimal temperature (probably not an issue for a mesophilic *Lactobacillus* species, but is for the *A*. *acidoterrestris* Cas12b/C2c1 enzyme, whose optimal temperature for activity is 50 °C), or sub-optimal biochemical conditions (ion composition and concentration in the mammalian environment being substantially different from that of the native bacterial host). The experiments presented here underscore the challenge of extracting potential new gene editing enzymes from native source bacteria and porting them to different eukaryotic hosts for use as molecular tools. While aspects of enzyme expression, ribonucleoprotein structure, or activity can be modulated, there is more work to be done both characterizing the variety of these CRISPR systems in their native organism as well as analyzing how context is different when required to be active inside the nucleus of a eukaryotic cell. Although outside the scope of the current study, the advent of efficient expression and purification (high yield and high purity) of newer Class II CRISPR effector enzymes, as well as the ability to synthesize guide RNAs for these systems, raises the possibility of addressing and possibly improving some of the limitations in nuclease activity. High resolution structural knowledge of this class of enzymes guiding an *in vitro* or *in vivo* (in bacterium) directed evolution assay might improve nuclease properties relating to issues such as guide RNA binding/loading, salt tolerance, DNA scanning and unwinding activity, temperature dependence, etc. If, through targeted mutagenesis, the basal level of nuclease activity could be improved in an *in vitro* system, perhaps the enzyme might have a better chance at gene editing when delivered to mammalian cells.

The *in vitro* cleavage assay using Lga Cas9 from cellular lysate was utilized in a high-throughput manner along with synthetic crRNA:tracrRNA guides to systematically test the PAM requirement of the enzyme. Bioinformatic analysis performed on productive CRISPR-Cas9 cut sites across a 1-base “walk” of 446 adjacent potential sites, agnostic of a PAM, in three different target gene amplicons, revealed that the most descriptive PAM for Lga Cas9 was NNRA/NDRA, or NNGA when allowing a flexible length distance between the PAM and the 20mer target sequence specified by the spacer region of the crRNA. This sequence is different than the NTAA PAM predicted for Lga Cas9 *in silico* by analysis of bacterial and phage/plasmid genomes. This requirement for Lga Cas9 effector nuclease cutting *in vitro* can be considered a target interference motif (TIM) versus a spacer acquisition motif (SAM), where a combination of the two motifs represents a true PAM in the native system [24]. It has been suggested that in some CRISPR systems a SAM is more restrictive than a TIM, and that differences in these motifs is dependent on the organism and the type of CRISPR system (Class I versus Class II). This is most likely due to differences in which overlapping sets of proteins are involved in the acquisition and interference steps of CRISPR immunity. In this case, it may be that the SAM, as predicted by the bacterial and phage genomes, is NTAA, where the less restrictive TIM as identified by this study is NNRA/NDRA, where the identity of second position is not required for interference and the third position preference is relaxed from A to a purine nucleotide (R, or G/A).

The concept of a PAM shifting by one nucleotide has been proposed for other Cas9 nucleases, at least in a subset of sequence contexts. While the predominant PAM for Spy Cas9 is NGG, a high-throughput PAM determination assay conducted in bacteria provided evidence that in a small number of sequence context-dependent cases NNGG is tolerated [8]. It is not known to what extent this positional flexibility of the PAM contributes to the PAM landscape for additional CRISPR-Cas9s or CRISPR systems generally. This factor should be considered when computing potential target sites, or more importantly *off-target sites*, of CRISPR effector nucleases for gene editing applications [25, 26].

Surprisingly, Lga Cas9 was also able, in several examples, to achieve productive cutting of target DNA with several adjacent 20mer crRNA target sites spaced by one nucleotide. This was unexpected considering that a PAM is considered to be determinate over several bases and to be located at a fixed distance away from the targeting/base-paired region where the crRNA binds (protospacer-*adjacent*).

Compared to the widely-used gene editing enzyme *S*. *pyogenes* Cas9 (whose PAM is NGG), a less restrictive PAM (NNRA/NDRA) or a flexible-length PAM for the Lga Cas9 enzyme (and potentially others) make these systems attractive for further exploitation in gene editing applications. Less restrictive PAMs, for instace those specifying only one determined nucleotide, would allow for a broader scanning range of potential target sequences, as more PAMs overall could be scanned for an adjacent 20-mer match targeting site. A 2-base determinant (such as NGG for Spy Cas) is present, on average, every 8 bases in the human genome, whereas a PAM such as NNRA can be found even more often [1].

*In vitro* investigation of both the functionality and the PAM preference of the Lga CRISPR-Cas9 system provides a framework to examine both challenges and opportunities when adopting a new CRISPR-Cas system for gene editing applications. There are many more systems awaiting discovery and characterization [27], as well as opportunities for enzyme evolution and engineering [7].

## Materials and methods

### Cloning of Cas9 expression vectors

The *L*. *gasseri* Cas9 gene was codon-optimized for human expression and synthesized (DNA2.0); its sequence is in S4 Table. Using sequence and ligation-independent assembly, it was cloned into a series of minimal mammalian expression vectors based on pUC18. Diagrams of the vectors are in S2 Fig.

### Protein expression Western blot analysis

HEK293T cells (obtained directly from ATCC, CRL-3216) were seeded in 6-well plates at 350,000 cells per well. The following day, Cas9 plasmids (5 μg) were lipofected (DharmaFECT Duo, 10 µL). After 48 hours, cells were harvested in a RIPA-based buffer supplemented with Protease Inhibitor cocktail (GE Healthcare, #80-6501-23). Protein concentration was estimated using the Pierce™ BCA Protein Assay (Thermo Fisher Scientific, #23225) as per the manufacturers protocol. 4xLDS (Thermo Fisher Scientific, #NP0008) was added to each lysate and was loaded onto a 4–20% gradient Tris-Glycine gel (Thermo Fisher Scientific, #EC6025BOX). Gels were run according to the manufacturers protocol, then proteins were transferred to nitrocellulose membranes (GE Healthcare Amersham, #10600107) at 30 V for 1 hour 45 minutes. After transfer, nitrocellulose membranes were blocked with SuperBlock Blocking Buffer (Thermo Fisher Scientific, #37515) for 30 minutes at room temperature. FLAG antibody (Sigma-Aldrich, #F1804) was used to detect Cas9 expression with PPIB antibody (Abcam, #ab16045) as loading control. Primary antibodies were diluted in SuperBlock and incubated overnight at 4° C. The next day, after washing, membranes were incubated with secondary goat anti-mouse antibody (Thermo Fisher Scientific, #32430), diluted in SuperBlock plus 0.05% Tween 20 at room temperature for 2 hours. After washing, western membranes were processed for ECL development using SuperSignal West Femto Maximum Sensitivity Substrate (Thermo Fisher Scientific, #34095) for FLAG and SuperSignal West Dura Extended Duration Substrate (Thermo Fisher Scientific, #34075) for PPIB as per the manufacturers protocol.

### Synthesis of tracrRNA and crRNAs

All crRNAs and tracrRNA were chemically synthesized on solid-phase support using 5ʹ-silyl-2ʹ-orthoester (ACE) chemistry [28, 29]. crRNAs were deprotected, desalted, and used without further purification. The tracrRNA was additionally HPLC-purified due to its longer length. The tracrRNA sequence and crRNA targeting sequences used are given in S4 Table.

### Preparation of whole-cell Cas9 lysate

HEK293T cells were seeded in 6-well plates at 500,000 cells per well. The following day, Cas9-expressing plasmid was lipofected into the cells (final concentration: 10 μL of DharmaFECT Duo transfection reagent, 5 μg of plasmid). The cells were incubated at 37° C with 5% CO_2_. After orty-eight hours, cells were washed once with PBS, trypsinized, and collected in a conical tube. After counting, they were spun down, washed with PBS, and resuspended at 10 milion cells in 500 μL in cold lysis buffer (20 mM Hepes pH 7.5, 100 mM KCl, 5 mM MgCl_2_, 1 mM DTT, 5% glycerol, 0.1% Triton X-100, and 1x cOmplete Protease inhibitor cocktail (Roche)); aliquots of cells were made in thin-walled PCR tubes (200 μL). Tubes of cells were sonicated in a cold water bath for 14 rounds of 30 seconds on, 30 seconds off. Cell lysates were combined, centrifuged at 14,000 rpm at 4°C, separated from cell debris and lysate aliquots were stored at -80° C until ready for use.

### *In vitro* lysate assay

Assays were performed using lysate prepared as described above. Cleavage assays were run in buffer (NEBuffer 3, 5 mM DTT, 10 mM MgCl_2_) for 1 hr at 37° C. Each reaction used 100–200 ng of target DNA and an equimolar ratio of crRNA:tracrRNA (400 nM, or 275 ng each). Target DNA consisted of genomic PCR amplicons from the EMX1, PSMD7, and VCP genes. Reactions were cleaned using PCR purification columns (QIAGEN) and run on 2% agarose gels containing ethidium bromide. Gels were visualized under UV light and imaged for band intensity using ImageJ (raw, digital images were used for quantitation).

### Co-transfection of Cas9 plasmid and crRNA:tracrRNA

HEK293T cells were seeded in 96-well plates at 20,000 cells per well. The following day, crRNAs and tracrRNA (GE Healthcare Dharmacon, #U-002000-50) were resuspended with 10 mM Tris-HCl pH 7.4 buffer (GE Healthcare Dharmacon, #B-006000-100) to 10 μM. For transfection in triplicate wells of duplicate cell plates, a deep-well plate was prepared with a 1:1 mixture of tracrRNA and each crRNA (5 μM each) diluted in MEM-RS medium (GE Healthcare HyClone, #SH30564.01) to 250 nM along with 1.4 μg of plasmid (70 μL total volume per well). DharmaFECT Duo transfection reagent (GE Healthcare Dharmacon, #T-2010-01) was diluted with MEM-RS medium (240 μL of DharmaFECT Duo transfection reagent was added to 3.76 mL of MEM-RS medium). Diluted transfection reagent (70 μL) was added to the crRNA:tracrRNA in the deep-well plates and incubated at room temperature for 20 minutes. After incubation, 560 μL of full growth medium was added per deepwell to obtain a complete transfection mixture (700 μL total) for triplicate transfection, in 2 plates. In each well, the medium on the plated cells was replaced with 100 μL of transfection mixture (final concentration: 0.6 μL of the DharmaFECT Duo transfection reagent, 200 ng of plasmid, and 25 nM of the crRNA:tracrRNA in each well). The cells were incubated at 37° C with 5% CO_2_ for 72 hours.

### T7EI mismatch detection (gene editing) assay

At 48 hours post-transfection, cells were lysed in a buffer containing proteinase K (Thermo Scientific, #FEREO0492), RNase A (Thermo Scientific, #FEREN0531), and Phusion HF buffer (Thermo Scientific, #F-518L) for 30 minutes at 56 °C, followed by a 5 minute heat inactivation at 95 °C. PCR was completed with primers flanking the cleavage site of each gene, after which reannealing of the PCR products was performed (See S4 Table for amplicon sequences). T7EI (New England Biolabs, #M0302L) was added to the PCR amplicons and incubated for 25 minutes at 37 °C to cleave mismatch strands. The T7EI cleavage products were separated on a 2% ethidium bromide-containing agarose gel. Gels were visualized under UV light and imaged for band intensity using ImageJ. The percent editing for each sample was estimated using the following calculation [1]:

$$\%\mathrm{indel}=(1-\sqrt{1-\frac{a+b}{a+b+c}})*100\%$$

*a*, *b*–intensity of cleavage product bands

*c*–intensity of uncleaved (wild type) band

### PAM walk design

A series of Lga crRNAs were synthesized walking across the 500–700 nucleotide PCR amplicon targets of the EMX1, PSMD7, and VCP genes, one base at a time. The series started 200 bases in from the 5 end and ended 200 bases from the 3 end, to allow visualization of the cut products on a 2% agarose gel. The amplicons and 20-mer target sequences of the crRNAs used are given in S4 Table.

### PAM walk analysis

We generated every permutation of PAM motif up to a length of 5 nucleotides. For each motif the experimental data were checked for how many crRNAs with and without the given motif showed cutting and did not show cutting. Those four counts for each motif were analyzed as 2x2 contingency tables using Fisher's exact tests by Microsoft Computational Biology Tools—False Discovery Rate application. The counts were also generated for each motif allowing for the motif to be shifted with respect to the end of the protospacer. The results of the Fisher's tests were then sorted and we report the most significant motifs.

## Supporting information

S1 Fig Western blot demonstrating that inker variation of FLAG-tag does not affect expression of Lga Cas9 species. Minimal expression plasmids encoding for hCMV promoter driven Lga Cas9 species were transfected in duplicate into HEK293T cells using DharmaFECT Duo. Plasmids contained no linker (LG08), a short linker (LG09) or long linker (LG10). As a positive control, a hCMV promoter driven Spy Cas9 was used. PPIB was used as a loading control.
(PPTX)

S2 Fig Schematic depiction of the various vectors designed for expression of Lga Cas9 protein (LGA03-LGA10) and Spy Cas9 protein (controls, SPY01- SPY03). The human CMV and CAG promoters were compared; the NLS was omitted or cloned on the N-terminus or C-terminus of Cas9 expressed from the CAG promoter, and finally the FLAG tag was placed on the N-terminus or C-terminus of Cas9 with various peptide linker lengths.
(PPTX)

S3 Fig A diagram demonstrating the experimental design of the *in vitro* PAM walk experiment. Three DNA amplicons were PCR-amplified from human genomic DNA for the three gene targts indicated: PSMD7, EMX1, and VCP (amplicon and crRNA target sequences are given in S4 Table). Lga Cas9 crRNAs were synthesized corresponding to 20mer targeting sequences “walked” across the amplicon sequences in 1-base increments, starting and ending 200 bases in from the 5’ and 3’ end of each amplicon. The number of crRNAs synthesized per amplicon is given, as well as the target site (underlined) and PAM (bold) for the positive control crRNA used for each amplicon.
(PPTX)

S1 Table Protospacers bioinformatically identified by alignment of spacers in the Lga CRISPR array to invading sequences (either phage or plasmid), as well as corresponding PAM sequences and the most closely representative PAM motif. (XLSX)

S2 Table The predicted Lga crRNA (targeting PSMD7) and tracrRNA sequences (wild type) were synthesized as shortened and lengthened forms for activity in an in vitro cleavage assay. (XLSX)

S3 Table crRNA target sites displaying activity (“hits”) in the PAM walk assay. (XLSX)

S4 Table Sequences of Cas9, amplicons, and crRNAs. (XLSX)
