# Supplementary figures and images for: Lactobacillus gasseri CRISPR-Cas9 characterization In Vitro reveals a flexible mode of protospacer-adjacent motif recognition
Source: PLoS One. 2018 Feb 2;13(2):e0192181. doi: 10.1371/journal.pone.0192181 (PMC5796720; doi:10.1371/journal.pone.0192181)

## Slide 1
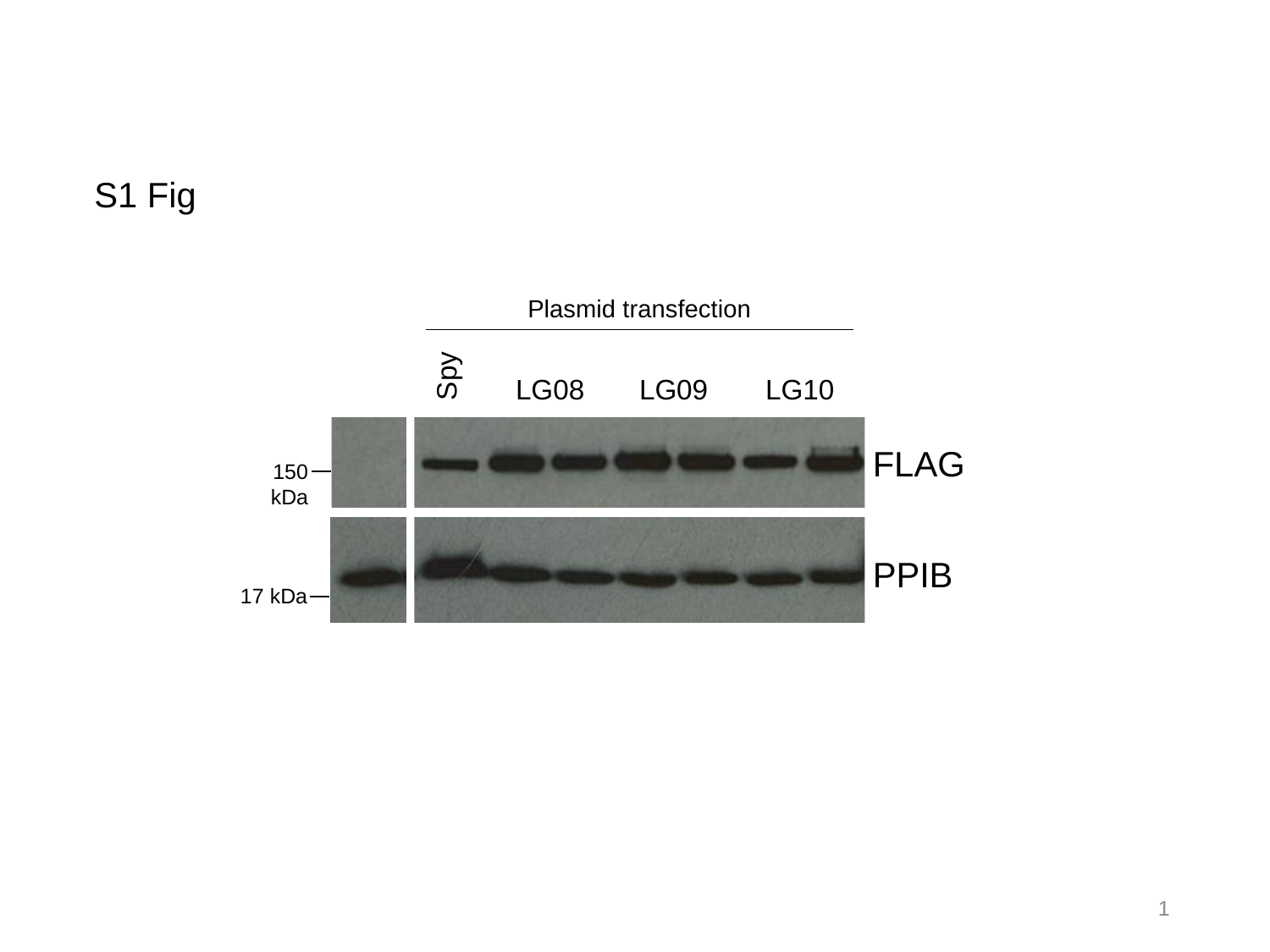

S1 Fig
Plasmid transfection
Spy
LG08
LG09
LG10
FLAG
150 kDa
PPIB
17 kDa
1

Supplement: S1 Fig — Minimal expression plasmids encoding for hCMV promoter driven Lga Cas9 species were transfected in duplicate into HEK293T cells using DharmaFECT Duo. Plasmids contained no linker (LG08), a short linker (LG09) or long linker (LG10). As a positive control, a hCMV promoter driven Spy Cas9 was used. PPIB was used as a loading control. (PPTX) [file pone.0192181.s001.pptx]
