# Supplementary material for: Lactobacillus gasseri CRISPR-Cas9 characterization In Vitro reveals a flexible mode of protospacer-adjacent motif recognition
Source: PLoS One. 2018 Feb 2;13(2):e0192181. doi: 10.1371/journal.pone.0192181 (PMC5796720; doi:10.1371/journal.pone.0192181)
Supplement: S2 Fig — The human CMV and CAG promoters were compared; the NLS was omitted or cloned on the N-terminus or C-terminus of Cas9 expressed from the CAG promoter, and finally the FLAG tag was placed on the N-terminus or C-terminus of Cas9 with various peptide linker lengths. (PPTX) [file pone.0192181.s002.pptx]

## Slide 1
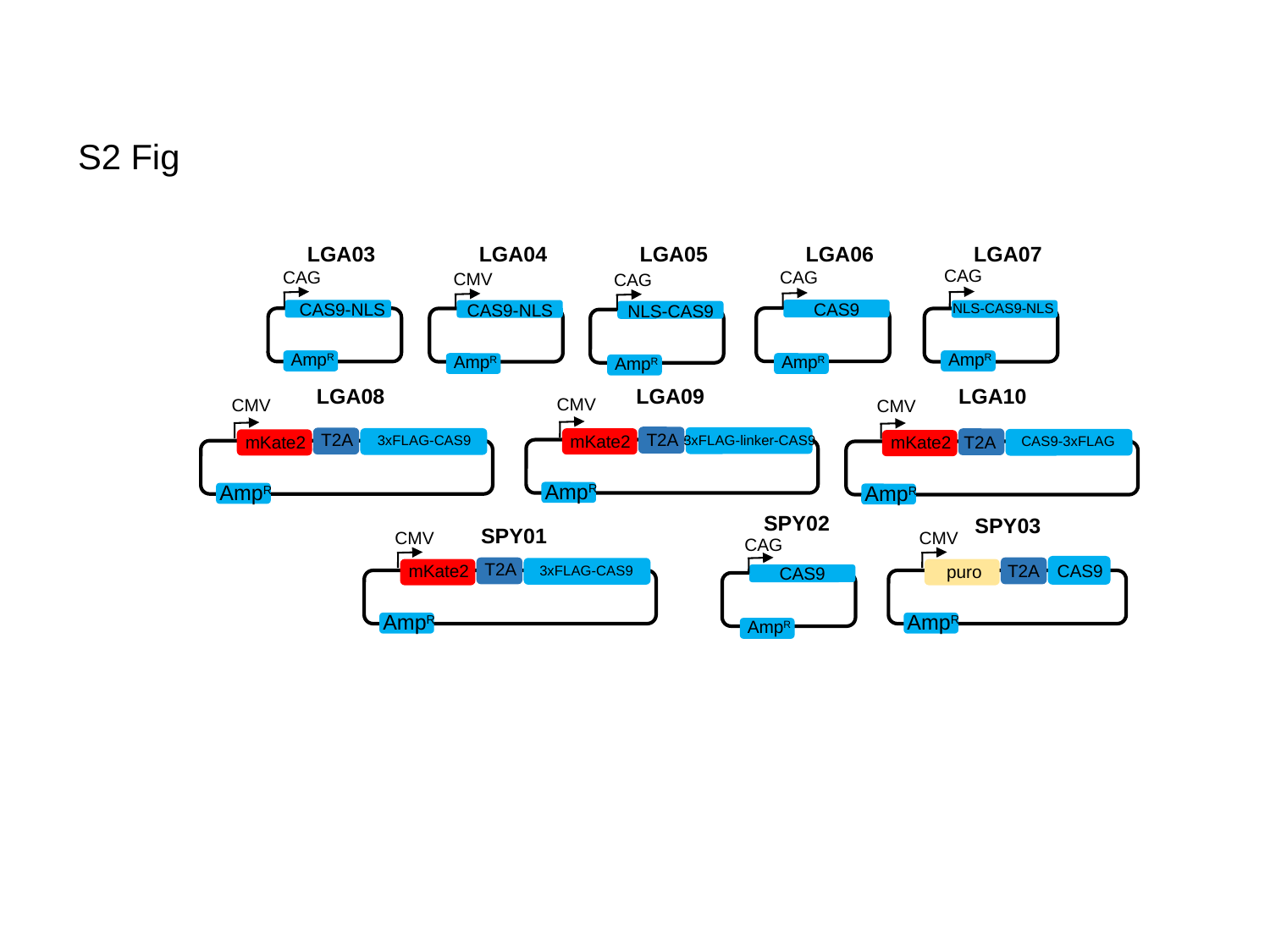

S2 Fig
LGA05
CAG
NLS-CAS9
AmpR
LGA06
CAG
CAS9
AmpR
LGA03
CAG
CAS9-NLS
AmpR
LGA04
CMV
CAS9-NLS
AmpR
LGA07
CAG
NLS-CAS9-NLS
AmpR
LGA09
CMV
T2A
mKate2
3xFLAG-linker-CAS9
AmpR
LGA10
CMV
T2A
mKate2
CAS9-3xFLAG
AmpR
LGA08
CMV
T2A
mKate2
3xFLAG-CAS9
AmpR
SPY02
CAG
CAS9
AmpR
SPY03
CMV
T2A
CAS9
puro
AmpR
SPY01
CMV
T2A
mKate2
3xFLAG-CAS9
AmpR
