# Supplementary material for: Lactobacillus gasseri CRISPR-Cas9 characterization In Vitro reveals a flexible mode of protospacer-adjacent motif recognition
Source: PLoS One. 2018 Feb 2;13(2):e0192181. doi: 10.1371/journal.pone.0192181 (PMC5796720; doi:10.1371/journal.pone.0192181)
Supplement: S3 Fig — Three DNA amplicons were PCR-amplified from human genomic DNA for the three gene targts indicated: PSMD7, EMX1, and VCP (amplicon and crRNA target sequences are given in S4 Table). Lga Cas9 crRNAs were synthesized corresponding to 20mer targeting sequences “walked” across the amplicon sequences in 1-base increments, starting and ending 200 bases in from the 5’ and 3’ end of each amplicon. The number of crRNAs synthesized per amplicon is given, as well as the target site (underlined) and PAM (bold) for the positive control crRNA used for each amplicon. (PPTX) [file pone.0192181.s003.pptx]

## Slide 1
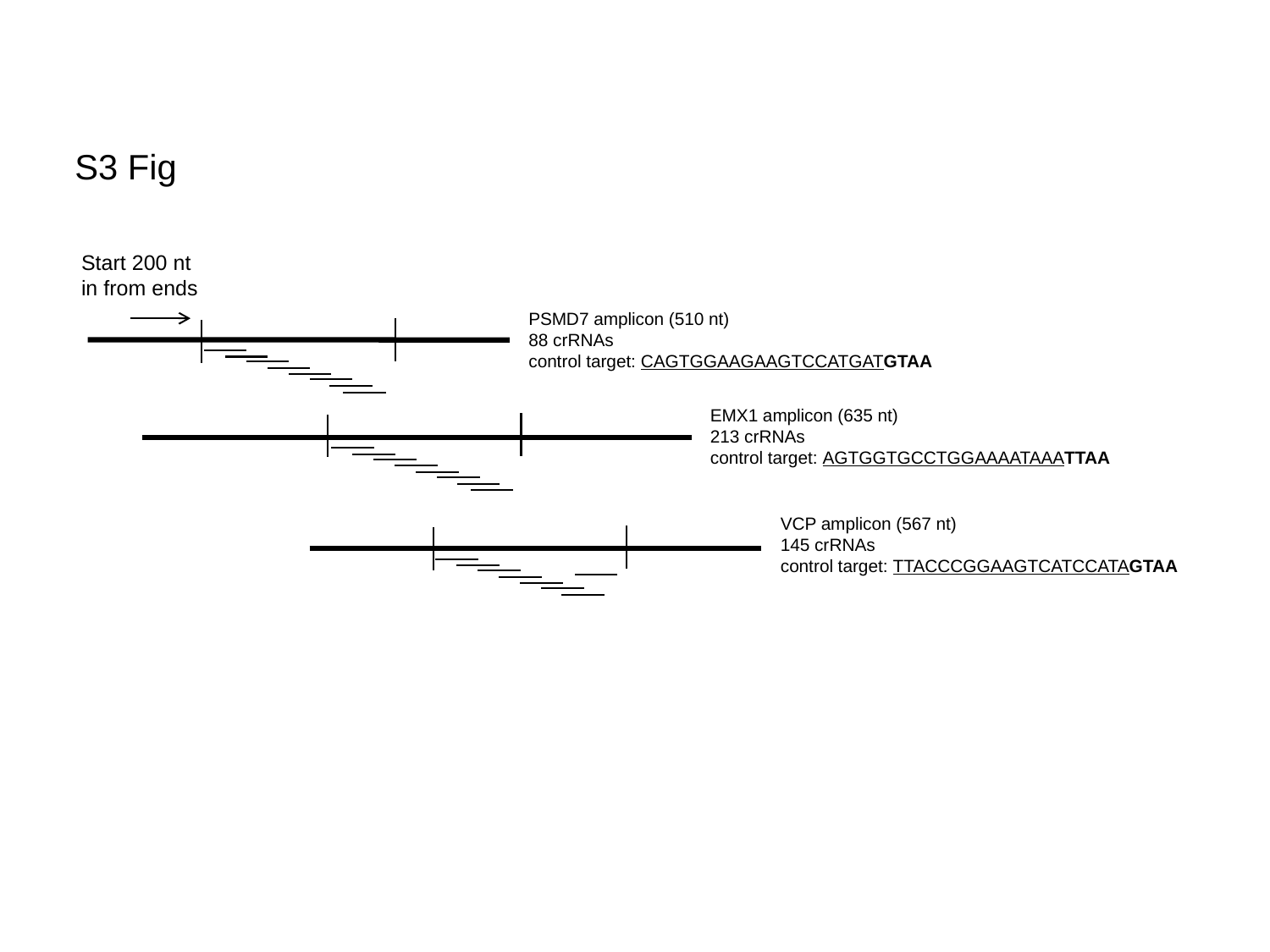

S3 Fig
Start 200 nt in from ends
PSMD7 amplicon (510 nt)
88 crRNAs
control target: CAGTGGAAGAAGTCCATGATGTAA
EMX1 amplicon (635 nt)
213 crRNAs
control target: AGTGGTGCCTGGAAAATAAATTAA
VCP amplicon (567 nt)
145 crRNAs
control target: TTACCCGGAAGTCATCCATAGTAA
